# Donor-Derived *Bartonella quintana* Infection in Solid Organ Transplantation: An Emerging Public Health Issue With Diagnostic Challenges

**DOI:** 10.1093/ofid/ofae381

**Published:** 2024-07-08

**Authors:** Carl Boodman, Oscar Fernandez Garcia, Dima Kabbani, Armelle Perez Cortes Villalobos, Amy Beeson, Grace E Marx, Johan van Griensven, Karen Doucette

**Affiliations:** Division of Infectious Diseases, Department of Internal Medicine, University of Manitoba, Winnipeg, Manitoba, Canada; Unit of Neglected Tropical Diseases, Institute of Tropical Medicine, Antwerp, Belgium; Department of Infectious Disease, Faculty of Medicine and Dentistry Medicine, University of Alberta, Edmonton, Alberta, Canada; Department of Infectious Disease, Faculty of Medicine and Dentistry Medicine, University of Alberta, Edmonton, Alberta, Canada; Division of Infectious Diseases, Department of Internal Medicine, University of Manitoba, Winnipeg, Manitoba, Canada; Division of Vector-Borne Diseases, Centers for Disease Control and Prevention, Fort Collins, Colorado, USA; Division of Vector-Borne Diseases, Centers for Disease Control and Prevention, Fort Collins, Colorado, USA; Unit of Neglected Tropical Diseases, Institute of Tropical Medicine, Antwerp, Belgium; Department of Infectious Disease, Faculty of Medicine and Dentistry Medicine, University of Alberta, Edmonton, Alberta, Canada

**Keywords:** bartonellosis, donor-derived infection, homelessness, lice, transplantation

## Abstract

*Bartonella quintana* is a louse-borne intracellular bacterium that remains a neglected cause of bacteremia, bacillary angiomatosis, and infective endocarditis among individuals experiencing poverty. In October 2023, Health Canada notified Canadian organ transplantation programs of an outbreak of donor-derived *B quintana* infection. From March to August 2023, 5 cases of donor-derived *B quintana* disease were acquired in Alberta, Canada, from 3 deceased donors who had experienced homelessness. Similar cases recently occurred in the United States. In this article, we discuss strategies to screen organ donors and monitor transplant recipients for *B quintana* infection using epidemiologic risk factors, physical examination signs, and laboratory diagnostic tests. We review the limitations of existing diagnostic tests for *B quintana* and describe how these problems may be magnified in the organ transplantation context.

## Teaser

In 2023, multiple cases of donor-derived *Bartonella quintana,* a louse-borne bacterium, were acquired in Canada and the United States from deceased donors who had experienced homelessness. We discuss strategies to screen organ donors and monitor transplant recipients for *B quintana* infection.

*Bartonella quintana* is a louse-borne intracellular bacterium causing bacteremia, bacillary angiomatosis, and infective endocarditis among individuals experiencing poverty [1–3]. The infection was first described in 1915 among World War I soldiers experiencing a relapsing febrile illness referred to as *trench fever*. In the 1990s, *B quintana* was determined to cause infective endocarditis and bacteremia among urban individuals experiencing homelessness in high-income countries: the term *urban trench fever* was coined [4, 5].

Transmission of *B quintana* involves the inoculation of infected human body louse feces into abrasions [1, 6]. Once in the host, the bacillus infects erythrocytes, causing chronic bacteremia that may last over a year despite minimal symptoms [3, 5]. As *B quintana* affects persons with body louse infestation (pediculosis corporis), the bacterial infection is closely linked to poverty, overcrowding, and inadequate access to running water and laundry services to maintain personal hygiene [3, 7, 8]. Species within the *Bartonella* genus, including *B quintana*, are challenging to culture and require longer than the routine 5-day incubation time [1]. *B quintana* infection is primarily diagnosed via serologic and molecular techniques [1].

In October 2023, Health Canada notified Canadian organ transplantation programs of an outbreak of donor-derived *B quintana* infection [9]. From March to August, 2023, 5 cases of donor-derived *B quintana* disease were acquired in Alberta, Canada, from 3 deceased donors [9]. Organ recipients presented with bacillary angiomatosis, fever, or polymorphic rash 4 to 10 months posttransplantation [9, 10]. All cases were molecularly confirmed to be *B quintana*, with the common risk factor being receipt of an organ from a donor who experienced homelessness within the 12 months prior to organ procurement [9, 10]. Retrospective testing of archived donor serum revealed positive serologies for *B quintana* [9].

Similar cases recently occurred in the United States: 2 kidney transplant recipients were infected with *B quintana* derived from a common donor experiencing homelessness [11]. One recipient was diagnosed with hepatic bacillary angiomatosis and vertebral osteomyelitis; a tissue sample was positive for *B quintana* according to molecular methods. The second recipient was asymptomatic, but active screening identified endocarditis with positive serology and peripheral blood positivity for *B quintana* by polymerase chain reaction (PCR). Residual tissue samples from the deceased donor tested positive for *B quintana* by molecular methods.

Previously, only 1 confirmed case of *B quintana* disease has been reported in a transplant recipient [12]. This individual presented with bacillary angiomatosis after receiving a kidney transplant in the Czech Republic, but donor history was not described. Due to the absence of *B quintana* risk factors among the recipients, these cases may be classified as probable donor transmission events [13]. All cases were unexpected by the transplantation teams; *B quintana* is not discussed in publications describing pathogens transmitted by solid organ transplantation [14].

In this article, we discuss strategies to screen donors and monitor recipients for *B quintana* infection. We review the limitations of existing diagnostic tests for *B quintana* and describe how these challenges may be magnified in the context of organ transplantation.

## DONOR SCREENING FOR *B QUINTANA* INFECTION

### Donor Risk Assessment According to Socioeconomic Risk Factors

As *B quintana* is primarily transmitted by body lice, specific populations are responsible for the majority of documented *B quintana* cases (Table 1). A personal history of body lice infestation suggests possible exposure to *B quintana*, as 17% to 33% of people with pediculosis corporis may be infected with *B quintana* [15]. Risk factors for *B quintana* infection include current or previous homelessness, history of living in a refugee camp or in a remote indigenous community without access to running water, and immigration from a low-income-country where overcrowding is common and access to running water is limited [1, 3]. While alcohol use disorder and male gender have historically been associated with *B quintana* infection, we suspect that these associations may be confounders, as both characteristics may be associated with homelessness [16]. Considering that *B quintana* bacteremia can often be chronic, with documented bacteremia lasting up to 8 years, risk factors for *B quintana* infection should include exposures that occurred in the past [5, 17]. The recent cases of donor-derived *B quintana* were all acquired from deceased donors who had experienced homelessness. Notably, the evaluation of epidemiologic risks for *B quintana*, such as a history of prior body louse infestation or housing instability, is often very challenging, and information obtained through medical record review may be incomplete. These cases emphasize the importance of obtaining past and current housing status for all patients and documenting this information in the medical record.

**Table 1.** Risk Assessment for Donor *Bartonella quintana* Infection

Risk factors for donor *B quintana* infection
Current or previous pediculosis corporis (body lice infestation) [1]
Current or previous homelessness [2, 3]
Current or previous use of shelter services (eg, clothing, meals, day programs)
History of living in a refugee camp [4]
Immigration from a low-income-country where access to running water is limited (risk may be increased in high-altitude areas such as the Ethiopian highlands) [5, 6]
Living on a Canadian indigenous community without access to running water [7]
Possible risk factors for donor *B quintana* infection
Injection drug use/death from drug overdose [1]
Significant alcohol use disorder [1]

References for Table 1 are listed in the Supplementary material.

### Physical Signs Suggestive of *B quintana* Infection Among Organ Donors

Physical assessment of the donor's body and clothing by the initial medical team may suggest *B quintana* infection through direct observation of body lice as well as dermatologic findings associated with pediculosis corporis, although we are aware that these examinations may be difficult for the organ procurement team to accomplish. Body lice, *Pediculus humanus humanus*, and their eggs (nits) may be visualized along clothing inner seams [6]. As body lice infestation causes significant pruritis, cutaneous signs include excoriations as well as postinflammatory hyperpigmentation. The inner lining of clothing may display blood stains. General signs of poor hygiene, such as heavily soiled clothing, may also be indicative of body lice exposure. While most donors infected with *B quintana* will likely have subclinical disease, direct signs of *B quintana* infection may include bacillary angiomatosis, heart failure, or the presence of a cardiac murmur [1].

### Donor Diagnostic Testing for *B quintana* Infection

Laboratory testing for *B quintana* involves different modalities, including specialized culture techniques, nucleic acid testing, and serology [18]. Each modality has its own limitations; thus, combination testing is often required to confirm a diagnosis. The slow turn-around time of *B quintana* testing combined with the expedited time of organ procurement precludes obtaining test results prior to organ transplantation.

Due to their intracellular localization, slow doubling time, and low bacteremia concentration in blood, species in the *Bartonella* genus, including *B quintana*, are not identified by routine blood culture with 5-day incubation [18]. While blood culture is recommended from deceased donors to identify potential donor-derived bacterial infections, these routine cultures are unlikely to isolate *B quintana*, as they do not employ prolonged incubation or specialized pathogen-specific methods [1, 14, 18]. Specialized techniques include methods to release the pathogen from its intraerythrocytic niche, such as freezing-thawing or lysis centrifugation, and prolonged microaerophilic incubation on Columbia blood-agar or shell-vial endothelial cell culture for multiple weeks [18]. However, even when these techniques are employed, sensitivity of blood culture may remain low: between 29% and 50% in cases of urban trench fever (defined as *B quintana* serologic positivity among homeless individuals with “symptoms of trench fever”) and endocarditis, respectively [18–20].

Nucleic acid amplification techniques, such as PCR, are mainstays of *B quintana* diagnosis with high specificity and the capacity to identify species within the *Bartonella* genus to the species level, when species-specific targets are used [21]. While the specificity of PCR testing from whole blood samples is excellent, its sensitivity remains low, between 33% and 58%, when tested in patients with proven *Bartonella* endocarditis [22]. This may be due to low bacterial quantity in the blood and the presence of PCR inhibitors such as hemoglobin and anticoagulants [18].

Serologic testing, most commonly by indirect immunofluorescent antibody assays (IFAs), provides a semiquantitative indication of current or previous *B quintana* infection [22]. IFA is associated with varying estimates of sensitivity depending on the assay, with 58% positivity documented among 106 cases of *Bartonella* endocarditis in one study, though other studies have reported higher sensitivity [19, 22]. IFA is also limited by a lack of specificity and known cross-reactivity with other pathogens [22]. Serology typically cannot identify *Bartonella* to the species level, though the National Reference Centre for Rickettsiosis in France combines Western blot with cross-adsorption techniques to compare serologic response to different *Bartonella* species [1]. Serologic titers >1:800 per the European Infection-Marseille serology (or 1:1024 with the DiaSorin/Focus IFA kit) are suggestive of infective endocarditis [23]. More recently, microbial cell-free DNA testing has emerged as a promising diagnostic method for *B quintana*, although this method is currently expensive and available at only 1 commercial laboratory.

Testing for *B quintana* is further complicated by organ transplantation. *Bartonella* testing often occurs at national reference laboratories with long turn-around times. Due to the limitations of each diagnostic test, organ transplantation programs may consider a combination of culture-based, serologic, and molecular tests for donors with identified risk factors for *B quintana*, such as donors with a history of homelessness, pediculosis corporis, or residence in a setting without access to running water to maintain personal hygiene (eg, a refugee camp). As *B quintana* may cause chronic bacteremia and *B quintana* disease may manifest years after initial infection, testing donors with previous risk factors may be considered, though data to guide the association between remote exposures and *B quintana* transmission are lacking [24]. Testing may include blood cultures with prolonged incubation; molecular testing based on PCR of whole blood samples and, if possible, blood vessel samples proximal to the transplanted organ (ie, *B quintana* has a tropism for erythrocytes and endothelial cells); and serology with IFA (Table 2). As the time frame of evaluation of deceased donors is typically hours, it is expected that organs will be transplanted prior to *B quintana* test results [14]. If a positive test result is identified prior to organ procurement, it does not preclude transplantation, as *B quintana* is treatable.

**Table 2.** Donor Diagnostic Testing for *Bartonella quintana* Infection

Test Category	Sample and Test Description	Comments
Bacterial culture	Blood culture samples Consider performing culture on donor spleen, if regularly extracted for other testing	Specialized techniques are required: Releasing pathogen from intracellular niche (eg, freeze-thaw, lysis centrifugation) [1, 2] Prolonged incubation in CO_2_-rich environment for up to 45 d [2] Consider addition of growth enrichment [3] Note: *Bartonella* species do not produce enough CO_2_ to trigger response from automatic culture systems [2]
Molecular	EDTA whole blood Consider performing on donor spleen or blood vessel samples proximal to donated organ	Molecular targets should include primers and probes specific to *Bartonella* genus (eg, ITS3) and *B quintana* species (eg, *fabB*, *yopP*, *groEL*) [1, 2, 4]
Serology	Serum for indirect immunofluorescent antibody test for both *B quintana* and *Bartonella henselae*; if serology for *B quintana* not available, proceed with serology for *B henselae* as cross-reactivity	Positive >1:100 (Infection-Marseille) or >1:124 (Focus/DiaSorin) Titers >1:800 (Infection-Marseille) or >1:1024 (DiaSorin/Focus Diagnostics) suggestive of infective endocarditis [5, 6]

References for Table 2 are listed in the Supplementary material.

## MONITORING RECIPIENTS AT ELEVATED RISK OF DONOR-DERIVED *B QUINTANA* INFECTION

### Recipient Risk Assessment for Donor-Derived *B quintana* Infection

Recipients at significant risk of donor-derived *B quintana* infection are those who receive organs from donors with active *B quintana* infection, as evidenced by direct detection of the bacterium by molecular or culture-based positivity on blood or tissue samples. Recipients of organs from donors with indirect detection of *B quintana* infection by serology may be at increased risk, although serology may indicate prior exposure with cleared or treated infection or cross-reactivity to other antibodies.

### Follow-up of Recipients at Risk for Donor-Derived *B quintana* Infection

Recipients with donor-derived *B quintana* infection may be asymptomatic or present with fever, culture-negative endocarditis, or a spectrum of other findings, including bacillary angiomatosis and osteomyelitis [9–11]. As such, we suggest that recipients who receive organs from donors with evidence of *B quintana* infection undergo evaluation with vital sign monitoring and complete physical examination with a focus on the dermatologic and cardiac examination (Table 3). Given the high risk of infection after organ receipt from donors with molecular or culture-based evidence of *B quintana*, a more intensive investigation or presumptive treatment may be considered. Recipients of organs from donors with positive serology but negative culture and molecular testing results may warrant less extensive monitoring. Recipients of organs from donors without known risk factors for *B quintana* are unlikely to be at significant risk; thus, no testing is suggested. Recipients of a donor whose other organ recipients tested positive for *B quintana* or developed symptomatic *B quintana* disease should also undergo more intensive monitoring, screening testing, and possible presumptive treatment.

**Table 3.** Suggested Monitoring and Interventions of Solid Organ Transplant Recipients at Elevated Risk of Donor-Derived *Bartonella quintana* Infection

Donor-Derived *B quintana* Infection	Within First Month^a^	3 mo	6–12 mo
High risk^b^	Physical examination PCR: EDTA whole blood Prolonged culture Indirect immunofluorescent antibody test Consider antimicrobial prophylaxis	Physical examination PCR: EDTA whole blood Indirect immunofluorescent antibody test	Physical examination PCR: EDTA whole blood Indirect immunofluorescent antibody test Transthoracic echocardiography^c^ Abdominal computed tomography scan or ultrasonography
Moderate risk^d^	Per high-risk recipients	Per high-risk recipients	Per high-risk recipients, though without radiographic investigations

For individuals with no known risk of donor-derived *B quintana* infection—specifically, no known history of donor risk factors, such as homelessness or pediculosis corporis—no monitoring is suggested. Note that no data about housing entails a lack of data, rather than no history of homelessness.

Abbreviations: IFA, immunofluorescent antibody assay; PCR, polymerase chain reaction.

^a^Within the first month or as soon as the donor results of *B quintana* testing are available.

^b^High risk of donor-derived *B quintana* infection: Donor has molecular or culture-based evidence of *B quintana* infection. Organ recipients from a donor whose other organ recipients tested positive for *B quintana* or developed symptomatic disease may be considered at elevated risk. Recipients of organs from donors with positive IFA results and no available *Bartonella* PCR and culture results may be considered at elevated risk.

^c^Echocardiography is to investigate for possible subclinical culture-negative infective endocarditis.

^d^Moderate risk of donor-derived *B quintana* infection: Defined as those with positive IFA results and risk factors for possible *B quintana* infection but negative culture and molecular testing results. Recipients of organs from donors with a documented history of homelessness or lice infestation and with no donor testing available may also be considered at moderate risk. Moderate-risk recipients may be monitored per recipients with high risk, though without the radiographic investigations at 6 to 12 months.

We suggest that, in addition to physical examination, periodic testing be considered by the organ transplantation team at 1 and 3 months for the recipient at elevated risk of donor-derived *B quintana* infection. Due to the sensitivity limitations of any 1 diagnostic test for *B quintana*, we also suggest testing for *B quintana* through a combination of molecular, serologic, and culture-based techniques (Table 3). Combination testing may identify early *B quintana* infection among recipients and therefore prevent possible severe outcomes, such as endocarditis or bacillary angiomatosis. Inflammatory responses associated with systemic bacterial infection are impaired by immunosuppressive medications among transplant recipients, which may result in reduced symptomatology and delayed presentation at an advanced stage of disease. The addition of molecular and culture techniques is recommended, as serologic testing such as IFA may be falsely negative or falsely positive in transplant recipients. Iatrogenic immunosuppression, needed to avoid graft rejection, compromises the sensitivity of immunologic testing, causing possible false negativity [25]. Conversely, antibody transfer from the donor may cause serologic false positivity among the recipients [26]. For recipients who received organs from donors with molecular or culture positivity for *B quintana*, transthoracic echocardiogram and computed tomography scans or abdominal ultrasonography may be considered between 6 and 12 months to investigate possible endocarditis, embolization, or hepatic bacillary angiomatosis, as cases of *B quintana* endocarditis may have an initial presymptomatic phase [3, 27]. As endocarditis and bacillary angiomatosis are chronic manifestations of *B quintana* that take time to develop, earlier radiographic investigations may not identify these severe syndromes. While data on transplant-derived *B quintana* are scant, all known cases manifested signs or symptoms within a year; as such, intermittent testing until 1 year posttransplant will likely identify most cases of donor-derived *B quintana* infection.

### Treatment of Recipients With Transplant-Derived *B quintana* Infection

Few data are available to guide decision making regarding treatment of transplant-derived *B quintana* infections. All 5 Albertan cases of transplant-derived *B quintana* infection reported by Health Canada were successfully treated with doxycycline, either as monotherapy or in combination with azithromycin [10]. The 2 organ recipients in the US cluster were successfully treated with similar regimens, with the addition of rifampin for 6 weeks for the patient with endocarditis [11]. The Czech patient was successfully treated with 3 months of doxycycline, followed by 3 months of clarithromycin [12].

Treatment of *B quintana* infection in the transplant recipient is complicated by interactions between anti-infective agents and immunosuppressants [28]. Many antimicrobials used in treating *B quintana* infection (rifampin and macrolides) interact with CYP3A4 (cytochrome 3A4), thereby modifying levels of iatrogenic immunosuppression. While doxycycline, the tetracycline backbone of systemic *B quintana* treatment, does not significantly interact with CYP3A4, rifampin induces the enzyme, decreasing immunosuppression caused by steroids, calcineurin inhibitors, and mammalian target of rapamycin inhibitors [28]. It is unknown whether rifabutin, a rifamycin that interacts less with immunosuppressants, may be used as a replacement for rifampin in severe *B quintana* infection. In cases of *B quintana* bacteremia and endocarditis, doxycycline is often combined with gentamicin [3]. The latter significantly enhances the nephrotoxicity of cyclosporin and tacrolimus [28]. Due to the pharmacokinetic complexities of treating *B quintana* infection in the transplant recipient, we suggest that health practitioners discuss cases of donor-derived *B quintana* infection with infectious diseases specialists, pharmacists, and the transplantation team to identify the optimal antimicrobial regimen and tailor the selection, dosing, and duration of antibiotic treatment and immunosuppressive medications, as required.

There are no data to guide decisions regarding antimicrobial prophylaxis vs preemptive therapy involving diagnostic monitoring at predefined intervals for recipients of organs from donors with documented *B quintana* infection. We suggest that the transplantation team discuss with the transplant recipient the risks and benefits of antimicrobial prophylaxis vs a preemptive therapy approach, which involves intermittent monitoring and waiting until evidence of *B quintana* infection is present before starting antimicrobial therapy. Patients with bacillary angiomatosis who are clinically stable may be treated with doxycycline or azithromycin monotherapy [29]. For patients with severe manifestations, including bacteremia and endocarditis, rifampin or gentamicin is often added as a second agent [29].

## DISCUSSION

The emergence of donor-derived *B quintana* disease likely reflects recent changes in organ transplantation and donor epidemiology, notably linked to the epidemic of drug overdose deaths and increased rates of houselessness [30]. Opioid overdose deaths in the United States have almost tripled in the last few decades, causing >100 000 deaths annually, and there has been a substantial increase in the transplantation of organs from donors who died of drug overdoses [30]. Transplantation of organs from overdose deaths has been facilitated by the availability of direct-acting antivirals for hepatitis C [31]. Transplantation of organs from donors who were hepatitis C positive and died of drug overdoses has increased dramatically in recent years [32]. While the link among opioid overdose, hepatitis C infection, and unstable housing is well established, housing status is rarely included in transplantation questionnaires [30, 33]. According to the US Department of Housing and Urban Development, 580 466 people in the United States experienced houselessness on a single night in 2020 with estimates that this figure may triple by 2030 [34]. In Canada, an estimated >235 000 Canadians experience houselessness in a given year [16].

Due to the link with pediculosis corporis, the recent increase in homelessness may partially explain the reemergence of *B quintana* infection in Canada and the United States. In 2020, Canada's largest cluster of *B quintana* infection was described among unstably housed persons in Winnipeg, Manitoba: 4 individuals required hospitalization for *B quintana* endocarditis within a few months [27]. In 2022, the first pediatric case of *B quintana* endocarditis acquired in a high-income country was reported from a remote Manitoban indigenous community with limited access to running water [8]. Prior to this outbreak, only 3 cases of *B quintana* infection were described in Canada [35]. Recent outbreaks of *B quintana* among houseless populations have also been documented in different jurisdictions in the United States, from Denver, Colorado, to New York City, New York, to Anchorage, Alaska [7, 36, 37].

The concurrent epidemics of drug overdose and homelessness coincide with recent changes in transplantation candidacy to likely contribute to the emergence of *B quintana* among solid organ transplant recipients. As *B quintana* is not a notifiable disease, the infection is likely underreported; thus, we recommend that transplantation programs identify alternate ways to track and report donor-derived transmission events due to *B quintana*. While characteristics of organ donors from overdose death have been published, the few articles on homelessness and organ donation remain in the realm of bioethics, rather than viewing homelessness as a risk factor for infectious disease transmission [30, 34]. We recommend that risk factors for *B quintana*, such as current or previous homelessness and pediculosis corporis, be included in donor questionnaires and their impact be evaluated in prospective studies. To facilitate a more comprehensive picture of donor-derived *B quintana,* risk factors and diagnostic testing for *B quintana* should be assessed via a standardized approach in prospective studies, under the guidance of national organ procurement organizations, such as the Organ Procurement and Transplantation Network [13]. We recommend that data on donor homelessness and transplant-related *B quintana* infection be reviewed by national disease transmission advisory committees, taking into consideration the nuanced definitions of housing status. These initial steps may improve our understanding of transplant-associated *B quintana* transmission events and clarify the burden and optimal management of this neglected infection among solid organ transplant recipients.

## Supplementary Data

Supplementary materials are available at *Open Forum Infectious Diseases* online. Consisting of data provided by the authors to benefit the reader, the posted materials are not copyedited and are the sole responsibility of the authors, so questions or comments should be addressed to the corresponding author.

## Supplementary Material

ofae381_Supplementary_Data
