# Supplementary material for: Donor-Derived Bartonella quintana Infection in Solid Organ Transplantation: An Emerging Public Health Issue With Diagnostic Challenges
Source: Open Forum Infect Dis. 2024 Jul 8;11(8):ofae381. doi: 10.1093/ofid/ofae381 (PMC11348938; doi:10.1093/ofid/ofae381)
Supplement: ofae381_Supplementary_Data [file ofae381_supplementary_data.docx]

**Appendix :**

References for table 1: Risk assessment for donor *B. quintana* infection:

1. Foucault C, Brouqui P, Raoult D. <em>Bartonella quintana</em> Characteristics and Clinical Management. Emerg Infect Dis J **2006**; 12:217. Available at: http://wwwnc.cdc.gov/eid/article/12/2/05-0874.

2. Spach DH, Kanter AS, Dougherty MJ, et al. Bartonella (Rochalimaea) quintana Bacteremia in Inner-City Patients with Chronic Alcoholism. N Engl J Med **1995**; 332:424–428.

3. Boodman C, Wuerz T, Lagacé-Wiens P, et al. Serologic testing for &lt;em&gt;Bartonella&lt;/em&gt; in Manitoba, Canada, 2010–2020: a retrospective case series. C Open **2022**; 10:E476 LP-E482. Available at: http://www.cmajopen.ca/content/10/2/E476.abstract.

4. Raoult D, Ndihokubwayo JB, Tissot-Dupont H, et al. Outbreak of epidemic typhus associated with trench fever in Burundi. Lancet **1998**; 352:353–358.

5. Angelakis E, Diatta G, Abdisa A, et al. Altitude-dependent Bartonella quintana Genotype C in Head Lice, Ethiopia. Emerg Infect Dis **2011**; 17:2357–2359.

6. Boodman C, Gupta N, Nelson C, van Griensven J. Bartonella quintana endocarditis: a systematic review of individual cases. Clin Infect Dis **2023**; Available at: https://doi.org/10.1093/cid/ciad706.

7. Boodman C, MacDougall W, Hawkes M, Tyrrell G, Fanella S. Bartonella quintana endocarditis in a child from Northern Manitoba, Canada. PLoS Negl Trop Dis **2022**; 16:e0010399. Available at: https://doi.org/10.1371/journal.pntd.0010399.

References for table 2. donor diagnostic testing for *B. quintana* infection

1. Foucault C, Brouqui P, Raoult D. <em>Bartonella quintana</em> Characteristics and Clinical Management. Emerg Infect Dis J **2006**; 12:217. Available at: http://wwwnc.cdc.gov/eid/article/12/2/05-0874.

2. Agan BK, Dolan MJ. Laboratory diagnosis of Bartonella infections. Clin Lab Med **2002**; 22:937–962. Available at: https://www.scopus.com/inward/record.uri?eid=2-s2.0-0036891905&doi=10.1016%2FS0272-2712%2802%2900017-3&partnerID=40&md5=41d8c8fab15c9649e6409b4b6e8137ac.

3. Wolf LA, Cherry NA, Maggi RG, Breitschwerdt EB. In Pursuit of a Stealth Pathogen: Laboratory Diagnosis of Bartonellosis. Clin Microbiol Newsl **2014**; 36:33–39. Available at: https://www.sciencedirect.com/science/article/pii/S0196439914000129.

4. Houpikian P, Raoult D. Blood culture-negative endocarditis in a reference center: Etiologic diagnosis of 348 cases. Medicine (Baltimore) **2005**; 84:162–173. Available at: https://www.scopus.com/inward/record.uri?eid=2-s2.0-18744370205&doi=10.1097%2F01.md.0000165658.82869.17&partnerID=40&md5=83be63b189261c3f0c85f439b80bebd3.

5. Edouard S, Nabet C, Lepidi H, Fournier P-E, Raoult D. Bartonella, a common cause of endocarditis: a report on 106 cases and review. J Clin Microbiol **2015**; 53:824–829. Available at: https://pubmed.ncbi.nlm.nih.gov/25540398.

6. Fowler Jr VG, Durack DT, Selton-Suty C, et al. The 2023 Duke-ISCVID Criteria for Infective Endocarditis: Updating the Modified Duke Criteria. Clin Infect Dis **2023**; :ciad271. Available at: https://doi.org/10.1093/cid/ciad271.
